# Radiological imaging of teratological fetuses: what can we learn?

**DOI:** 10.1007/s13244-017-0551-8

**Published:** 2017-04-24

**Authors:** Lucas L. Boer, A. N. Schepens-Franke, J. J. A. van Asten, D. G. H. Bosboom, K. Kamphuis-van Ulzen, T. L. Kozicz, D. J. Ruiter, R-J. Oostra, W. M. Klein

**Affiliations:** 1 Department of Anatomy and Museum for Anatomy and Pathology, Radboud University Medical Centre, Geert Grooteplein Noord 21, 6525 EZ Nijmegen, The Netherlands; 2 Department of Radiology and Nuclear Medicine, Radboud University Medical Centre, Nijmegen, The Netherlands; 3 Department of Anatomy, Embryology and Physiology, Academic Medical Centre, University of Amsterdam, Amsterdam, The Netherlands

**Keywords:** Education, Magnetic resonance imaging, Computed tomography, Teratology, Congenital anomaly

## Abstract

**Objectives:**

To determine the advantages of radiological imaging of a collection of full-term teratological fetuses in order to increase their scientific and educational value.

***Background﻿*:**

Anatomical museums around the world exhibit full-term teratological fetuses. Unfortunately, these museums are regularly considered as “morbid cabinets”. Detailed dysmorphological information concerning the exhibited specimens is often lacking. Moreover, fetuses with severe and complex congenital anomalies are frequently diagnosed incompletely, incorrectly or not at all.

**Methods:**

In order to verify diagnoses and to enrich their educational and scientific value, we imaged 41 out of the 72 teratological specimens present in the collection of our Anatomy and Pathology Museum in Nijmegen (The Netherlands) by means of magnetic resonance imaging (MRI) and computed tomography (CT). Additionally, contemporary dysmorphological insights and 3D models are implemented in the teratology education of medical students and residents.

**Conclusions:**

Full-term teratological fetuses have become increasingly rare and deserve a prominent place in every anatomical museum; they are suitable for contemporary teratological research and education. Modern radiological techniques markedly enhance their scientific and didactic value.

***Teaching Points*:**

• *To explore the scientific and educational potential of institutionalised teratological collections*

• *To understand the additional value of radiological imaging in diagnosing teratological specimens*

• *To learn about the specific settings of MRI parameters when scanning fixed specimens*

• *To recognise specific internal dysmorphology in several congenital anomalies*

## Introduction

Many anatomical museums around the world exhibit teratological specimens of third trimester fetuses. Among the institutionalised collections, especially noteworthy are the eighteenth century collection of the Federal Pathological Anatomy Museum in Vienna (Austria) [1, 2], the eighteenth century collection of the Hunterian Museum of the Royal College of Surgeons in London [3] and the nineteenth century Vrolik collection residing in the Vrolik Museum at the University Medical Centre of Amsterdam [4]. They all contain a rich trove of teratological specimens. Although some academic institutions have abandoned their anatomical collections because of apparent legal issues, safety reasons, financial cuts or newly defined priorities, these museums are much more than time capsules with accumulations of curiosities [5]. They can be regarded as vibrant, inspirational, instructive and interdisciplinary academic working environments with scientific and educational potentials that can be exploited in (bio)medical curricula or in resident training programs [6]. However, one might wonder if anatomical museums should still exhibit full-grown dysmorphic fetuses as these types of anomalies are rarely occurring events in modern times. Moreover, one could question whether historical teratological specimens still have a contemporary value in a period of daily evolving medical innovations and molecular technology. These are issues anatomical museums have to deal with on a daily basis [7, 8].

Congenital anomalies have intrigued mankind since the earliest times. Already in ancient cultures terracotta ornaments were fabricated depicting congenital anomalies. These were clearly based upon existing cases, indicating that they were perceived as divinities, omens or even punishments of supernatural origin [9, 10]. Nowadays, people with very diverse backgrounds visit teratological collections residing in medical museums. Knowledge of both the normal and abnormal embryological development is important for both teachers and physicians while medical students and patients have a constantly growing medical knowledge and ask more sophisticated questions [6]. This implies that a teratological collection can be of great value to educate people about human development. Although several anatomical museums expose teratological specimens, most institutions lack detailed external and internal (dys)morphological descriptions or imaging. Because of this, collections are often stigmatised as “morbid cabinets”.

The teratological collection of the Museum for Anatomy and Pathology in Nijmegen, The Netherlands, currently possesses 72 specimens. It was collected by Albert Verhofstad (deceased 2008), who was affiliated to the Radboud University Medical Centre in Nijmegen, between the 1950s and 1970s. The collection originates from before the ubiquitous availability and utilisation of (high-resolution) ultrasound for prenatal screening and therefore most specimens are full-grown fetuses or newborns. Nowadays full-grown fetuses with severe congenital anomalies are rarely born in well-developed countries. This implies that teratological collections become more valuable with time.

**Fig. 1:**
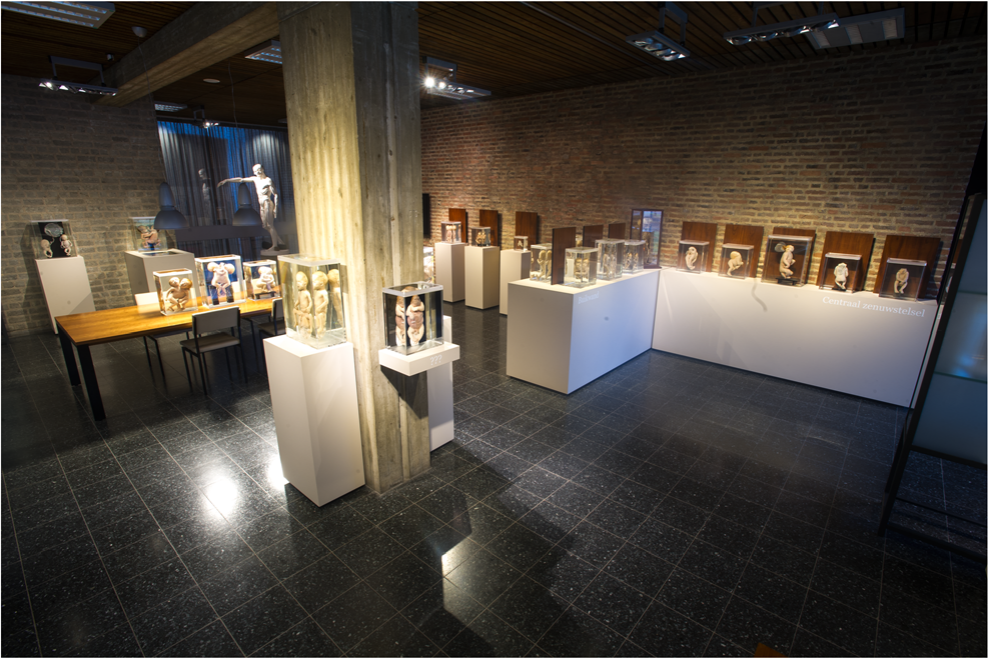
Photograph of the teratological collection in the Museum for Anatomy and Pathology of the Radboud University Medical Centre in Nijmegen, The Netherlands. The exhibit displays 35 teratological fetuses, 10 specimens of animal teratology, historical books on teratology, 3D models and plaster casts

In the past, our collection of teratological fetuses too was often seen as a “morbid cabinet” by both students and the general public. In the exhibition, there was neither a clear choice of the exhibited fetuses nor was there any systematic approach recognisable; questions about the nature and pathogenesis of several congenital anomalies could not be answered. Therefore, in order to systematically expose the teratological collection of our museum, we defined nine anomaly groups, into which all 72 specimens could be categorised. This categorisation led to an new exhibition, in which 35 of the most educational specimens found a permanent expository position (Fig. 1).

**Fig. 2:**
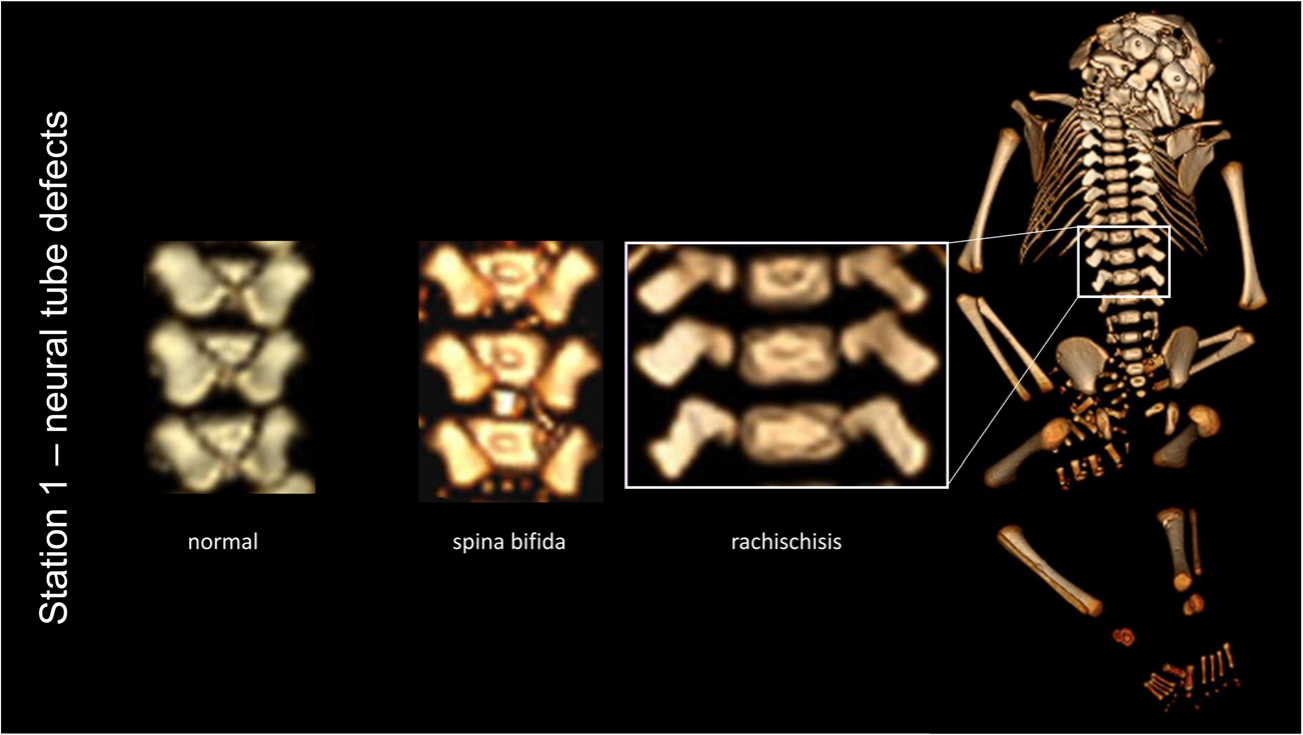
Screenshot of the interactive information of neural tube defects using radiological imaging: vertebrae of a fetus with rachischisis can be compared to vertebrae of a normal fetus and a fetus with spina bifida

In order to increase the scientific and educational value of the specimens of this unique teratological collection, we wanted to elucidate the internal (dys)morphological characteristics. Instead of invasive exploration, computed tomography (CT) and magnetic resonance imaging (MRI) techniques were used to generate detailed images of the internal (dys)morphology of the teratological fetuses. Radiological imaging proved to be an excellent method to investigate these delicate specimens in a non-invasive manner [11]. Recently, the museum opened an innovative exhibition of specimens documented with these images. This exhibition is accessible for both medical students and the general public. Radiological imaging and information about normal development and pathogenesis can be obtained by consulting a touchscreen in which all specimens are described in full detail (Fig. 2). Common information is given on physical billboards throughout the exposition.

Here we report on the radiological imaging results and we describe four fetuses (cases 1–4), in which new diagnoses or interesting morphological characteristics were established. Furthermore, we discuss the scientific and educational benefits that can be gained from radiological imaging of dysmorphic fetuses. The purpose of this paper is to present an approach to create an innovative teratological exposition in order to de-stigmatise and to more profoundly educate the (bio)medical student and professional.

## Materials and methods

The entire collection of 72 teratological fetuses was visually inspected and re-described according to contemporary syndromological views by a panel of experts in 2012 (R.J.O., A.N.S.F. and L.L.B.). Verification of the identified syndromes and sequences was obtained by consulting peer groups and contemporary handbooks on clinical syndromology [12, 13]. Several anomaly groups were defined according to the classification of congenital anomalies described by the European Surveillance of Congenital Anomalies [14]. Radiological imaging was used to generate detailed images of the internal (dys)morphology. An inclusion criterion for radiological imaging was that the specimen had not been previously subjected to autopsy in the area of interest. Furthermore, the group with neural tube defects consisted of 20 fetuses. From these fetuses, six fetuses were selected: three that displayed iniencephaly and three with a variety of other neural tube defects. This resulted in the radiological imaging of 41 specimens. Radiological imaging consisted of a total body MRI and total body CT; scanning protocols are described below. Prior to scanning, the specimens were taken out of their jars, thoroughly rinsed with demineralised water and placed in disposable plastic bags to prevent dehydration during imaging. After imaging, the specimens were replaced in a 4% formaldehyde solution. Radiological data were reviewed by three radiologists with expertise in paediatric neurological (K.K.v.U.), cardiothoracic (D.G.H.B.) and abdominal/musculoskeletal (W.M.K.) radiology, all with previous expertise in fetal post-mortem imaging.

### MRI protocol

Specimens were scanned on a TIM TRIO 3-T MRI scanner (Siemens, Erlangen, Germany). Specimens smaller than 30 cm in length were placed in a standard circular head and neck coil, specimens over 30 cm in length were measured with an additional body coil. MRI scan parameters of the clinical fetal post-mortem MRI were transformed to optimise the spatial resolution of the specimens as they had stayed in a 4% formaldehyde solution for 50–60 years [15, 16]. An overview of the MRI parameters is given in Table 1.

**Table 1:** Summary of the MRI sequence parameters

Sequence	Voxel (mm^3^)	Fov (mm)	TE (ms)	TR (ms)	NA	Flip angle (°)	TA(min)
T1w flash 3D	∼0.5 × 0.5 × 0.5	300–400	5	11–13	6–8	25	90
T2w TSE 3D (SPACE)	∼1.2 × 0.7 × 0.5	300–400	184–479	3,280	4–9	Var exc.	30–45

*Fov* field of view, *TE* echo time, *TR* repetition time, *NA* number of averages, *flip angle* radiofrequency power, *TA* total acquisition time, *T1w* T1-weighted, *T2w* T2-weighted, *TSE* turbo spin echo

### CT protocol

The specimens underwent a total-body CT scan in a CT scanner (Aquilion One Vision Edition, Toshiba, Japan). Two protocols were used to cover the different sizes of the specimens; one volume scan mode for fetuses smaller than 16 cm and one helical scan mode for fetuses larger than 16 cm. Three-dimensional (3D) reconstructions were made using a filter convolution (FC) of 30 for bone and a FC of 07 for soft tissue, both with adaptive iterative dose reduction (AIDR) in 3D. An overview of the CT protocol parameters is listed in Table 2.

**Table 2:** CT parameters

Protocol	Potential (kV)	Current (mA)	Rotation (s)	∆Slice (mm)	Increment (mm)	Pitch	Collim. (mm)
Scan <16 cm	80	300	1	0.5	0.25	0 (vol.)	scanlen∙0.5
Scan >16 cm	80	400	1	0.5	0.25	0.813	80 ∙ 0.5

## Results

We performed CT and MRI scanning on 41 of the 72 teratological specimens. The radiological imaging had no effects on the condition of the specimens and no complications in the specimen conservation were discovered. CT and MR images were found to be of very high quality. Although we encountered some problems with post-mortem artefacts, e.g. shrinkage of the brain, decalcified skeletons and unusable radiographic skeleton surveys, most radiological data were of sufficient quality to re-diagnose and describe the internal characteristics of each fetus. An overview of the scanned fetuses is given in Table 3. We give an extensive description of a selection of four cases below.

**Table 3:** Diagnostic revision in 41 scanned teratological fetuses

Anomaly group	Previous diagnosis	Diagnosis after radiology
Ventral body wall defects	- ventral body wall defect with cleft lip and encephalocele - ventral body wall defect with neural tube defect - ventral body wall defect (3×)	- amniotic band sequence with concomitant ectopia cordis, unilateral CLP and unilateral temporal encephalocele - vascular disruption sequence with concomitant occipital encephalocele and gastroschisis - OEIS complex with concomitant omphalocele - OEIS complex with concomitant gastroschisis and ambiguous genitalia - OEIS complex with concomitant gastroschisis and spina bifida
Skeletal dysplasias (osteochondrodysplasias)	- achondroplasia (3×)	- thanatophoric dysplasia type I (*case 1*) - osteogenesis imperfecta type II (*case 2*) - short-rib polydactyly syndrome; not otherwise specified
Congenital teratomas	- teratoma	- oropharyngeal teratoma/epignathus
Conjoined twins	- conjoined twins (9×)	- cephalothoracoileopagus - prosopo-ileopagus - thoracoileopagus tribrachius - thoracoilieopagus tetrabrachius - ischiopagus tripus - ischiopagus tetrapus - diprosopus tetrophthalmus diotis with concomitant craniorachischisis totalis - parapagus dicephalus dibrachius dipus (*case 3*) - craniopagus
Syndromes with multiple congenital anomalies	- syndrome (2×) - phocomelia	- Meckel-Gruber Syndrome - bilateral schisis (most likely trisomy 13) - tetra-amelia syndrome (*case 4*)
Sirenomelia	- sirenomelia (7×)	- isolated sirenomelia type I (3×) - isolated sirenomelia type II - VACTERL association with concomitant sirenomelia type II - VACTERL-H association with concomitant sirenomelia type I - VACTERL-H association with concomitant sirenomelia type II
Holoprosencephaly	- cyclopia (6×)	- alobar HPE (4×) - alobar HPE with concomitant otocephaly (2×)
Neural tube defects	- iniencephaly (3×) - occipital encephalocele/exencephaly - craniorachischis - craniorachischis totalis	- iniencephaly - iniencephaly with concomitant semi-lobar HPE and omphalocele - iniencephaly with concomitant myelomeningocele - occipital encephalocele/exencephaly - craniorachischis - craniorachischis totalis
Unknown specimen	- unknown diagnosis	- unknown diagnosis

*CLP* cleft lip and palate, *OEIS* omphalocele-exstrophy-imperforate anus-spinal defects, *VACTERL* vertebral defects, anal atresia, cardiac defects, tracheo-oesophageal fistula, renal anomalies and limb abnormalities, *VACTERL-H* vertebral defects, anal atresia, cardiac defects, tracheo-oesophageal fistula, renal anomalies and limb abnormalities with hydrocephaly, *HPE* holoprosencephaly

### Skeletal dysplasias

**Fig. 3:**
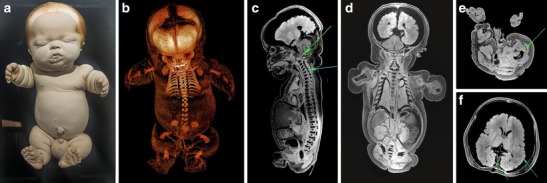
**a** Photograph of the full-term fetus of case 1. **b** Three-dimensional reconstructed skeleton based on the CT data. Although there was severely diminished bone calcification, CT images visualised extremely shortened ribs, short and small scapulae and iliac bones, and femoral and humeral bowing. **c** Sagittal T1-weighted MR image which showed a small foramen magnum with slight cranio-cervical caudal transition (*green arrow*), platyspondyly of the vertebra with short vertebral arches resulting in spinal canal stenosis (*turquoise arrow*). **d** Coronal T1-weighted MR images showed a severely hypoplastic thorax with presumably severe lung-hypoplasia. **e** Transverse T1-weighted MR image on the level of the femoral heads showed broad and irregular metaphyseal plates and extreme femoral bowing (*green arrow*) sometimes referred to as “telephone receiver” femora. **f** Transverse T1-weighted MR image of the brain which showed courser gyri of the temporal lobes (*turquoise arrow*) and excessive gyration of the occipital lobes (*green arrow*) and can be interpreted as polymicrogyria. Note the shrunken brain and small lungs which were both interpreted as normal post-mortem artefacts and probably strengthened by the formalin fixation. However, the presence of lung hypoplasia cannot be ruled out

Case 1 concerns a full-term, large, male stillborn, which showed on external examination a disproportionate micromelic shortening of all extremities, a narrow “bell-shaped” thorax, protuberant abdomen and relatively large scrotum. All extremities showed redundant skin folds with severe brachydactyly and mildly affected trident hands. Craniofacial abnormalities included macrocephaly, severe frontal bossing, prominent cheeks and chin, ocular proptosis, a depressed nasal bridge and a severe hypoplastic midface with hypertelorism, a prominent tongue and slightly recessed ears (Fig. 3a). The former diagnosis of this specimen, before re-examination in 2012, was achondroplasia. The CT images revealed that the calcification of the bones was severely diminished (Fig. 3b). MRI images did reveal the contours of the bones. Based on the combination of the micromelic shortening of all extremities, bowing of both femora, short ribs, bell-shaped thorax with small chest cavity and presumably hypoplastic lungs, platyspondyly of the vertebra and the polymicrogyria we diagnosed the condition as thanatophoric dysplasia (TD) type I (Fig. 3b–f). Neonatal death was most likely due to respiratory insufficiency and/or compression of the spinal cord or brainstem by spinal stenosis.

**Fig. 4:**
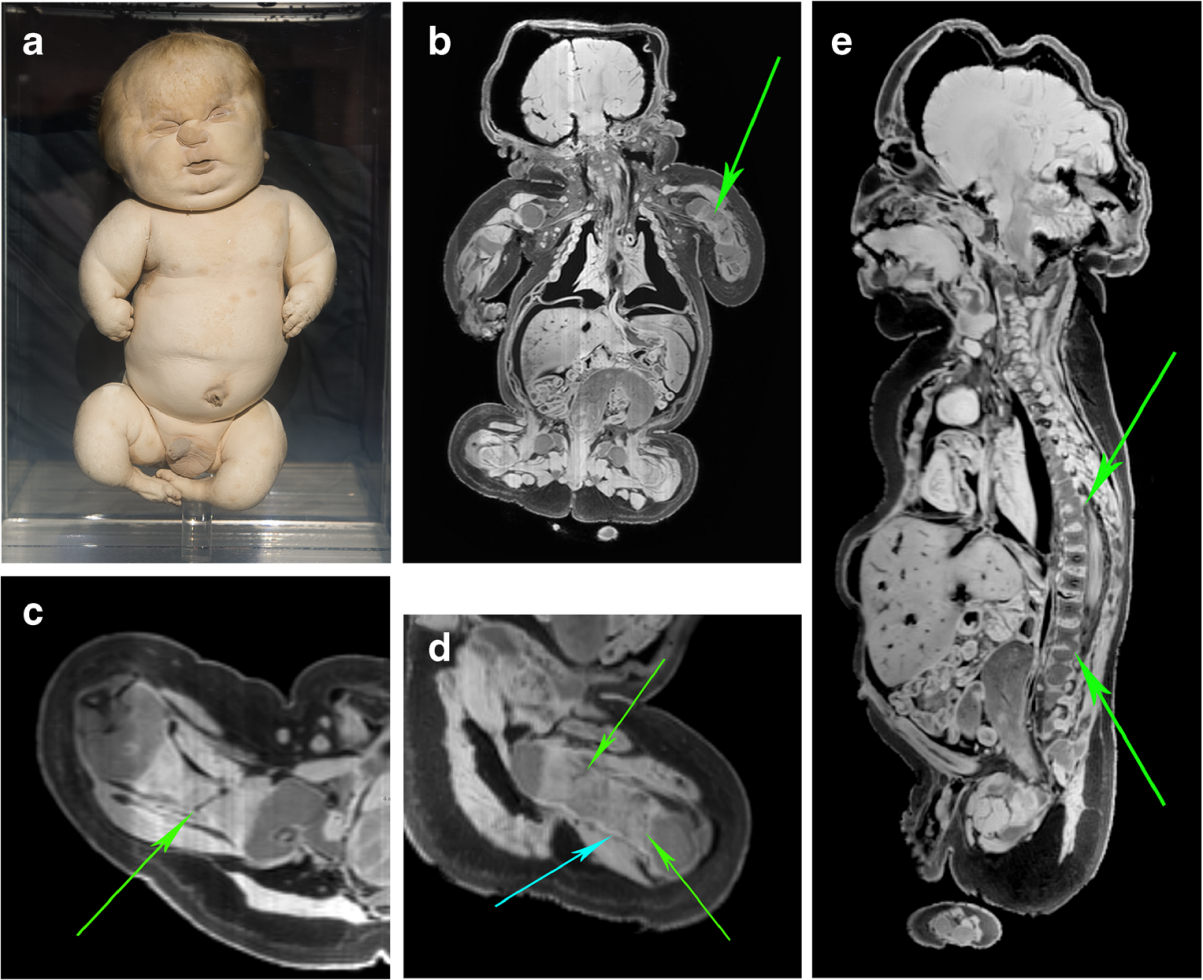
**a** Photograph of the full-term fetus of case 2 demonstrating a characteristic facial appearance, protruding abdomen and severely bowed extremities. **b** Coronal T1-weighted MR image showed an aberrant and irregular humerus (*green arrow*). Note the severely shrunken lungs insight the hypoplastic thorax; interpreted as shrinkage of the lungs due to post-mortem artefacts and formalin fixation. However, the presence of lung hypoplasia cannot be ruled out. **c** Transverse T1-weighted MR image of the broadened and shortened right femoral bone which showed a fracture (*green arrow*). **d** Transverse T1-weighted MR image of the severely shortened and aberrant left femoral bone which showed multiple fractures (*green arrows*) and irregular cortical bone (*turquoise arrow*). **e** Sagittal T1-weighted MR image showed multiple fractures in the vertebral column (*green arrows*). Note the distorted calvarium due to limited mineralisation and the shrunken brain due to the formalin fixation

Case 2 concerns a full-term, male neonate, which showed on external examination a protruding abdomen, excessive bowing of all extremities and mesomelic shortened arms. Craniofacial abnormalities included a hypoplastic midface, microstomia, recessed ears and a somewhat flattened face. The head appeared to be positioned directly on the thorax with absence of the neck. The lower extremities were positioned in a frog-like position (Fig. 4a). The diagnosis of this specimen, before re-examination in 2012, was achondroplasia. The calcification of the bones was severely diminished, resulting in non-diagnosable CT images (not shown). MR images, however, did reveal the contours of the bones. Based on the distinct presence of multiple prenatal fractures, poorly mineralised and deformed cranial vault, secondarily healed osseous structures and small chest with presumably lung hypoplasia (Fig. 4b–e) the diagnosis osteogenesis imperfecta (OI) type II was made with reasonable certainty.

### Conjoined twin

**Fig. 5:**
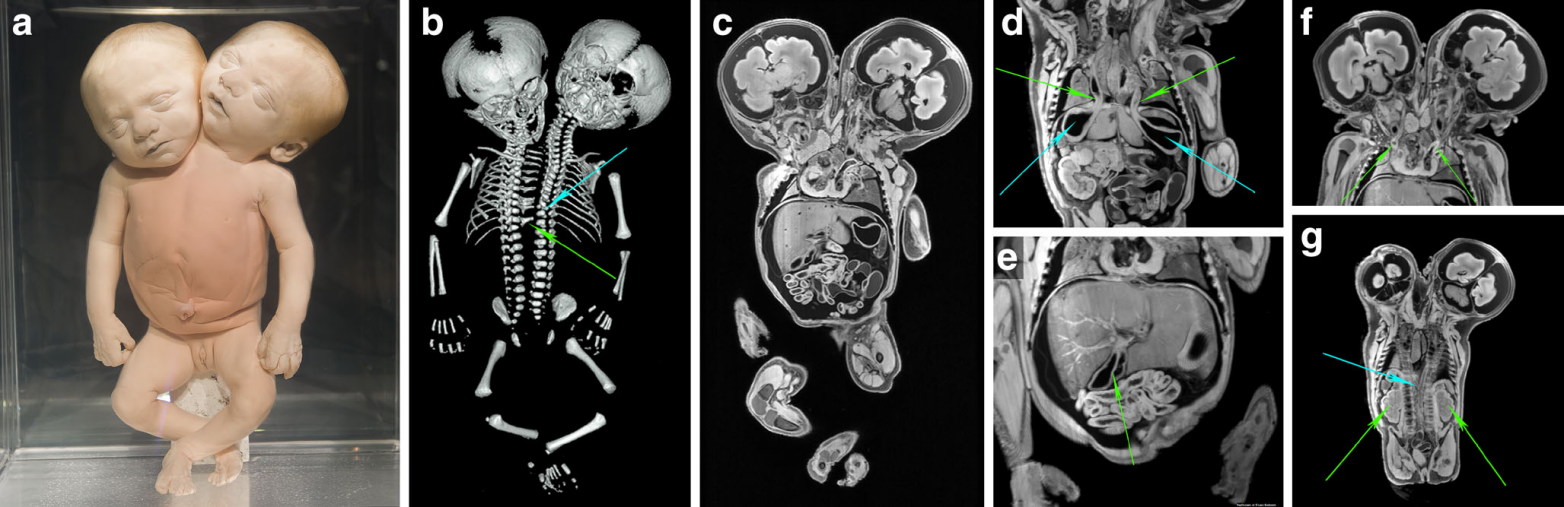
**a** Photograph of the full-term conjoined twin of case 3. **b** Three-dimensional reconstructed skeleton based on the CT data showed butterfly and block vertebra (*turquoise arrow*) with fused ribs (*green arrow*) between the two separate vertebral columns There were two heads, two arms and two legs with one broad pelvis. **c** Coronal T1-weighted MR image showed two normal brains, one shared “fused” heart and liver with one overarching diaphragm. **d** Coronal T1-weighted MR image showed two separate oesophagi (*green arrows*) and two separate stomachs (*turquoise arrow*). **e** Coronal T1-weighted MR image showed a “fused” liver with two gallbladders (*green arrow*). **f** Coronal T1-weighted MR image showed two ascending aortas (*green arrows*). **g** Coronal T1-weighted MR image showed two kidneys and adrenal glands (*green arrows*) and one anus. The two descending aortas fused at the level of the 11th thoracic vertebra (*turquoise arrow*). Note that the right descending aorta is smaller than the left descending aorta

**Fig 6:**
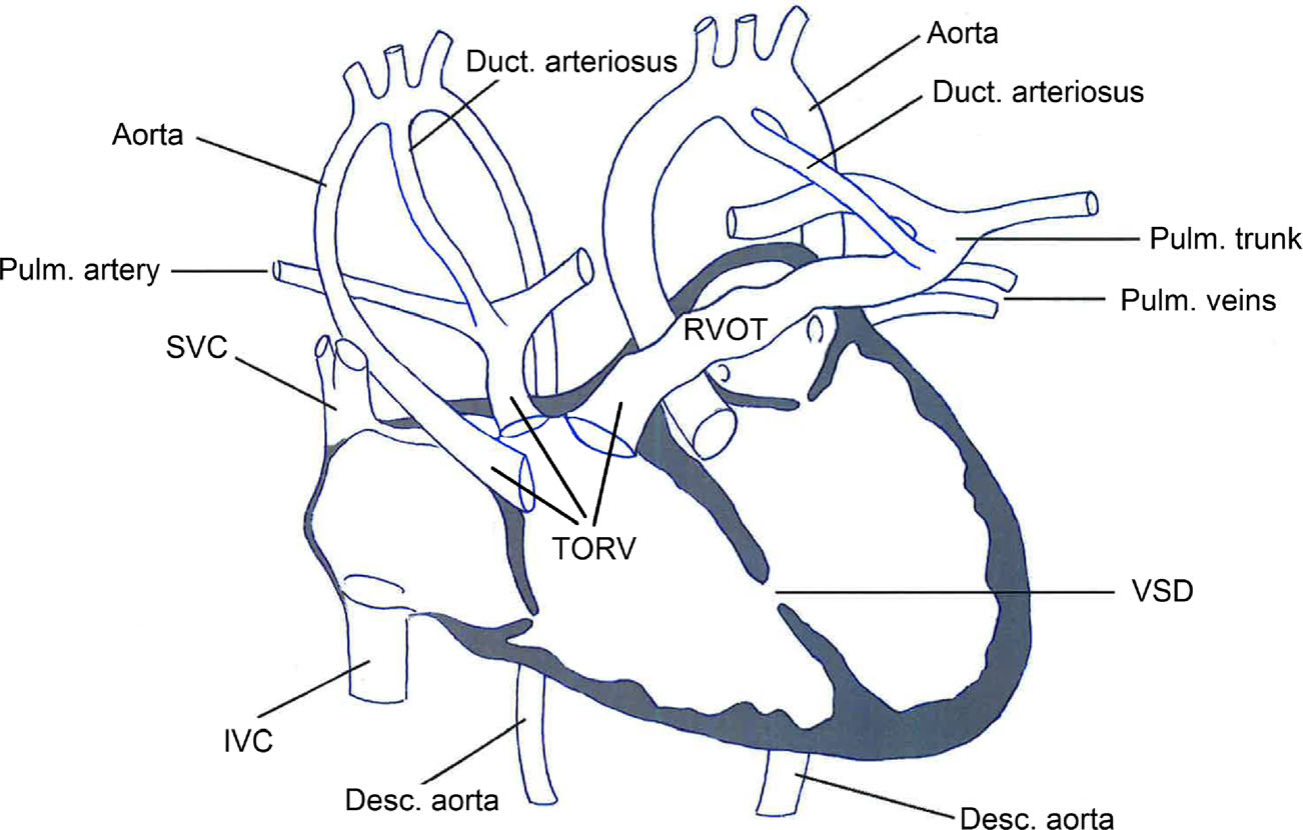
Schematic drawing of the fused heart of case 3. There were two ventricles and two atria. A normally located left aorta arose from the left ventricle. At the right atrium small pulmonary veins were seen. From the right ventricle the second, relative smaller, right aorta, right pulmonary trunk and a right ventricle outflow tract (*RVOT*) of the left pulmonary trunk were seen; this could be interpreted as a triple outflow right ventricle (*TORV*). There were two ducti arteriosi. On the right side no clear pulmonary veins were seen. There was a ventricular septal defect (*VSD*) resulting in a complex haemodynamic situation

Case 3 concerns a small, full-term, female, conjoined twin with two heads, two arms and two legs (Fig. 5a). The diagnosis of this specimen, before re-examination in 2012, was conjoined twin. After re-describing and imaging this fetus, we diagnosed this specimen as parapagus dicephalus dibrachius dipus (*see* “Discussion”). Both CT (Fig. 5b) and MR (Fig. 5c–g) data were of excellent quality to describe the intricate internal dysmorphological characteristics. Additionally, based on the MRI data, a schematic drawing was made of the morphology of the “fused” heart in order to get insight into the complex haemodynamic situation (Fig. 6).

### Tetra-amelia syndrome

**Fig. 7:**
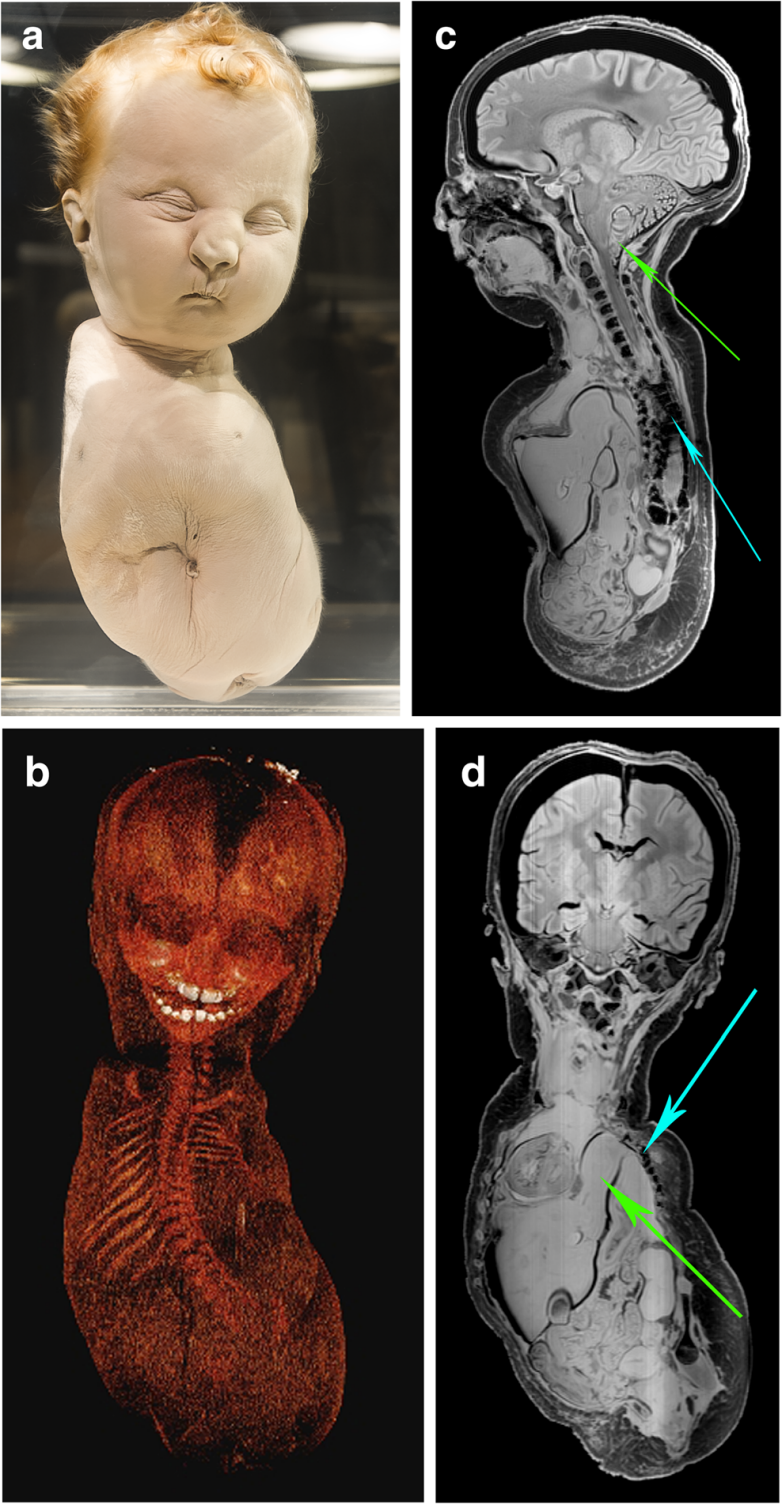
**a** Photograph of the full-term fetus of case 4. **b** Three-dimensional reconstructed skeleton based on the CT data. Although there was severely diminished bone calcification, CT images visualised small pelvic bones, partial absence of the sacral bone and a thoracolumbar convex shaped curvature. The scapulae and clavicles were normally developed with absence of the humeral, ulnar and radial bones including the hands. In addition, the femora, tibial and fibular bones including the feet were absent. **c** Sagittal T1-weighted MR image showed an Arnold-Chiari malformation (*green arrow*) and distorted vertebral column (*turquoise arrow*). **d** Coronal T1-weighted MR image showed a severe diaphragmatic hernia (*green arrow*) which deviated the heart completely to the right lateral thoracic wall. The left pleural cavity was almost entirely occupied by the liver, stomach, spleen and left adrenal gland, the left lung was merely noticeable. Moreover, a distortion of the left ribs was seen (*turquoise arrow*)

Case 4 concerns a full-term, female neonate, which showed on external examination total absence of all four limbs, micrognatia, microstomia, mild Potter’s facies and hypertelorism. A left-oriented deviation of the relatively small body is noticeable (Fig. 7a). The diagnosis of this specimen, before re-examination in 2012, was phocomelia. The CT images reveal that the calcification of the bones is severely diminished (Fig. 7b). MR images reveal the contours of the bones (Fig. 7c and d). Additionally, we found a concomitant diaphragmatic hernia, skeletal anomalies and a Arnold-Chiari malformation. We diagnosed the condition as tetra-amelia syndrome with a concomitant diaphragmatic hernia; a rarely described association [17].

## Discussion

Although many anatomical museums display teratological fetuses on a smaller or larger scale, these displays usually lack comprehensive pathogenetic storylines, additional radiological imaging of the exposed specimens, and most importantly, they often neglect their potential value in biomedical curricula. Furthermore, the diagnoses that fetuses bear are often incomplete, incorrect or outdated. As we demonstrated here, radiological imaging combined with contemporary dysmorphological knowledge was in most cases valuable or even essential to arrive at the correct diagnosis and to unveil the internal and sometimes unexpected peculiarities. Nowadays, many congenital and inherited anomalies can be diagnosed genetically. However, embalmed museological specimens frequently have fragmented and contaminated DNA, which is unsuitable for genetic exploration of candidate genes. We tried molecular inversion probe (MIP) techniques for targeted sequencing of genomic regions with potential candidate genes of multiple fetuses; unfortunately, without satisfactory results.

Cases 1 and 2 concern two distinct skeletal dysplasias, or osteochondrodysplasias, most of which originate from genetic defects that cause aberrant histological formation, growth and maturation of osseous and/or cartilaginous tissues. They usually affect all skeletal elements equally, leading to a decreased postural length (dwarfism). Therefore, skeletal dysplasias can be seen as generalised qualitative disorders of the skeleton, without primarily affecting the body plan [18]. Although achondroplasia is a specific diagnosis among the more than 300 skeletal dysplasias presently known, it has long been used as a generic term for any type of skeletal dysplasia, as it was in the cases described here. Despite the decalcification of the skeleton, which was probably largely caused by decalcification of the bone tissue due to the acidification of formalin through time [19], radiological imaging made it possible to diagnose TD type I in case 1 and OI type 2 in case 2. TD is genetically related to (true) achondroplasia but it is much more severe, whereas OI is caused by a genetic defect in collagen formation, which leads to (extremely) brittle bones. The imaging results demonstrate the pathogenesis, severity and potential lethality of the conditions in these cases, which markedly adds to their didactic value.

Case 3 concerns a pair of conjoined twins. Despite being a rare congenital malformation with an incidence of 1:200,000 live births and 1:200 monozygotic twins, it is a widely known phenomenon among scientists and laymen alike [20]. For many centuries, multiple rather enigmatic pathogenic hypotheses have been postulated, none of which satisfactorily explains their pathogenesis and conjunctional morphology. An intriguing, though not undisputed theory was postulated by Spencer in 2003 [21]. Her model hypothesises the presence of two (instead of one) embryonic primordial discs “floating” on the surface of a shared yolk sac (resulting in ventral and lateral conjunction types) or on a shared amniotic cavity (resulting in dorsal/neural conjunction). This “spherical coalescence” theory therefore postulates a secondary, symmetrical or asymmetrical, homologous conjunction of initially separate embryonic discs and subsequent embryonic fusion. The nature and extent of the conjunction result from the initial reciprocal distance and position of the two primordial discs on the yolk sac or amniotic cavity. Case 3 concerned a parapagus dicephalus dibrachius dipus conjoined twin, which can be concluded from external dysmorphological findings. However, radiological imaging revealed the intricate internal morphology and conjunction of organs, such as the heart and liver in this specific type, which is essential to understand the pathogenesis of conjoined twinning.

Finally, case 4 presented with tetra-amelia syndrome: an extremely rare disorder characterised by the absence of all four limbs. Infants are often stillborn or die perinatally due to lung hypoplasia and concomitant anomalies such as microstomia and micrognathia. No estimates on prevalence are described due to its rarity. After radiological imaging, we found a diaphragmatic hernia in concomitance with tetra-amelia: this is only rarely found and scarcely described in the modern literature [17, 22]. Although, diaphragmatic hernia is atypical in tetra-amelia syndrome, the acquired images can be used to educate the medical students on the subjects of congenital diaphragmatic hernias and the secondary effect on thoracic organ development.

The most convincing argument for the radiological imaging of a collection of teratological fetuses is the dramatic increase of internal dysmorphological insight obtained in a non-invasive manner. Although many teratological fetuses can be diagnosed and used in an educational setting based on their external dysmorphological appearance, radiological imaging increases the diagnostic value immensely in specifying anomaly subtypes (e.g. in sirenomelia), re-diagnosing anomalies (e.g. skeletal dysplasias) or in teaching certain embryologically oriented pathogeneses (e.g. conjoined twins). Moreover, radiological findings can strengthen arguments regarding pathogenetic hypotheses and thus lead to new or improved insights.

Because of currently available prenatal screening options, pregnancies complicated by congenital anomalies are often terminated well before full-term development. Nowadays, stillborn fetuses in general, let alone fetuses with rare congenital anomalies, are almost never assigned to scientific body donation programs. This results in an absence of supplementing teratological collections, which makes historical specimens of teratological full-term fetuses increasingly valuable and irreplaceable.

We posit that when well-defined teratological specimens are displayed respectfully with additional pathognomonic storylines and radiological data, these exhibitions are educationally legitimate and instructional for any museum visitor. The acquired radiological data are essential to educate the student and the resident on the subject of teratology. Additionally, these high-resolution radiological images can be used to help the obstetrician to recognise congenital anomalies during prenatal screening. Radiological techniques transform the “old and dusty” anatomical museums into modern academic and dynamic working environments suitable to educate the student as well as the (paediatric) radiologists in training. Moreover, radiological imaging of teratological collections makes students wonder and enthusiastic about the use of radiology in their curriculum and learn to compare images with the observed (museological) specimen. Finally, radiological findings can strengthen arguments regarding embryologically oriented pathogenetic hypothesises. By imaging and re-diagnosing teratological specimens that display a similar condition congenital anomalies can be studied beyond the limitations of single case studies and the spectrum or heterogeneity of a congenital anomaly becomes more clear. Therefore, we conclude that teratological collections are a treasure chest for radiologists, paediatricians, geneticists, pathologists and embryologists, and are of interest for additional (re)describing and imaging following new imaging techniques.

## Conclusions

Teratological specimens are becoming increasingly rare and deserve a prominent place in anatomical museums. These collections are very suitable for contemporary teratological research and can be used for public and medical education. As shown in this paper, radiological imaging is essential to reveal all the diagnostic ins and outs of old teratological specimens.
